# Feigning ADHD and stimulant misuse among Dutch university students

**DOI:** 10.1007/s00702-020-02296-7

**Published:** 2021-01-13

**Authors:** Anselm B. M. Fuermaier, Oliver Tucha, Janneke Koerts, Lara Tucha, Johannes Thome, Frank Faltraco

**Affiliations:** 1 Department of Clinical and Developmental Neuropsychology, University of Groningen, Grote Kruisstraat 2/1, 9712 TS Groningen, The Netherlands; 2 Department of Psychiatry and Psychotherapy, University of Rostock, Rostock, Germany

**Keywords:** Adult ADHD, University, Feigning, Medication, Stimulant misuse

## Abstract

The increasing number of university students seeking diagnosis of attention-deficit/hyperactivity disorder (ADHD), and findings of an increased stimulant misuse among university students, has raised concerns regarding the credibility of the symptoms of those students. However, most of our current knowledge refers to university students in North America and less is known about this issue on European campuses. The present survey aimed to collect opinions on feigning ADHD and to estimate the prevalence of stimulant misuse among 1071 university students in the Netherlands. The majority of students expressed liberal attitudes towards feigning ADHD. Also, a substantial number of respondents considered feigning ADHD themselves or know someone who feigns ADHD. Furthermore, 68% of students assumed benefits of taking stimulants without prescription and 16% have indeed already taken stimulants without prescription. Feigning ADHD and misuse of prescription medication are prevalent issues among Dutch students. The results underline the need for a careful diagnostic evaluation of individuals for ADHD. Furthermore, efforts are required in order to prevent stimulant drug trafficking and misuse among university students.

## Introduction

Attention-deficit/hyperactivity disorder (ADHD) is a neuropsychiatric developmental disorder which affects about 1.2–7.3% of adults worldwide (Fayyad et al. 2007). ADHD impacts negatively on multiple aspects of daily living, including learning and achievements in the educational setting (Daley and Birchwood 2010; Barkley et al. 2007), which is a matter of concern since ADHD was reported to be present in about 4% of college students (Rostain 2006).

However, research in the last decades also revealed high base rates of invalid symptom reports and performances among college students that are clinically evaluated for ADHD. Studies on university students in the United States and Canada demonstrated that about 15–48% of all college students that are presented for ADHD assessment show indications of exaggerated or feigned symptoms (Marshall et al. 2016; Sullivan et al. 2007; Harrison and Edwards 2010). One of the primary motives to feign ADHD was reported to be the easy access to stimulant medication that is commonly prescribed for the treatment of ADHD (Wigal 2009; Rabiner 2013). Stimulant misuse is a prevalent issue among college students, with rates of 6.9% of students in the United States (McCabe et al. 2006), and 6.6% of students in Canada (Poulin 2007). Other estimates go even up to 15% of all students in the United States that are assumed to have used stimulants without prescription (Rostain 2006; Rabiner 2013; Teter et al. 2013). The widespread misuse of stimulants is supported by findings showing that 26% of those students prescribed with stimulants gave or sold it to others on their university campus (Poulin 2007). Prevalence rates of stimulant misuse among students in Europe appear to be lower than in the USA or Canada, however, findings are difficult to compare because of differences in study design, study population, and specific questions asked in the surveys (Franke et al. 2014; Schelle et al. 2015).

Despite the clear evidence on the occurrence of feigned ADHD and misuse of prescription stimulants among university students in North America, less is known about the relevance of this issue on European campuses. The present study aimed to address this topic on a large sample of Dutch university students. A survey was performed asking about opinions on and experiences with feigned ADHD and stimulant misuse, including motives for taking prescription stimulants. Conclusions can be drawn regarding the susceptibility of the validity of psychiatric evaluations of university students for ADHD, and the need for prevention efforts of stimulant drug trafficking and misuse at European universities.

## Methods

### Participants

One thousand and seventy-one students from the social sciences of the University of Groningen, the Netherlands, took part in the study and completed the survey (Table 1). Because of the topic of this survey on feigning ADHD and misusing stimulant medication, students were not considered for inclusion if they indicated to be diagnosed with ADHD (*n* = 42). Thus, the remaining sample of 1071 individuals did not contain any students who reported to be diagnosed with ADHD. The vast majority of students were in their first year. Moreover, females took part in the study in larger numbers than males. The self-rated study performance of respondents was mixed ranging from very bad (1) to very good (5). None of the respondents reported to be diagnosed with ADHD.

**Table 1:** Characteristics of participants

Total *N*	1071
Age (in years, M ± SD)	20.3 ± 2.3
Gender (f/m)	780/291
Study year (1st/2nd/3rd/ ≥ 4th)	956/68/27/20
Study performance (M ± SD)^a^	3.57 ± 0.78

^a^Ranging from very bad (1) to very good (5)

### Materials and procedure

The present study was part of a larger survey on university students. To learn about attitudes towards and experiences with feigning ADHD and stimulant misuse, only a selection of items was used for the present context, i.e. whether (1) *they ever considered to feign ADHD* (Yes/No), (2) *they know someone who feigns ADHD* (Yes/No), (3) *they think it is easy to feign ADHD* (Yes/No), and (4) *they think there are benefits to feign ADHD* (Yes/No), including the *type of benefits* (open response format). Further items were analyzed asking respondents whether (5) *they think there are benefits to take stimulants (as usually prescribed for the treatment of ADHD) without prescription* (Yes/No), and whether (6) *they have ever taken stimulants (as usually prescribed for the treatment of ADHD) without prescription* (Yes/No), including *frequency of use*, *source of medication*, as well as their *motives* for either taking (multiple answers in categories possible) or not taking stimulants (open response format).

The study was approved by the ethical committee psychology (ECP) affiliated to the University of Groningen, the Netherlands. The survey was to be filled in online and took about 20 min to complete. All respondents were informed about the purpose of the study beforehand and gave consent by clicking on a specified button at the introductory page that prompted participants to the start of the survey. It was made explicit to participants that data were stored and processed anonymously. Participation was voluntary and not paid. However, first-year psychology students were awarded with study credits as part of their undergraduate research requirement.

## Results

**Fig. 1:**
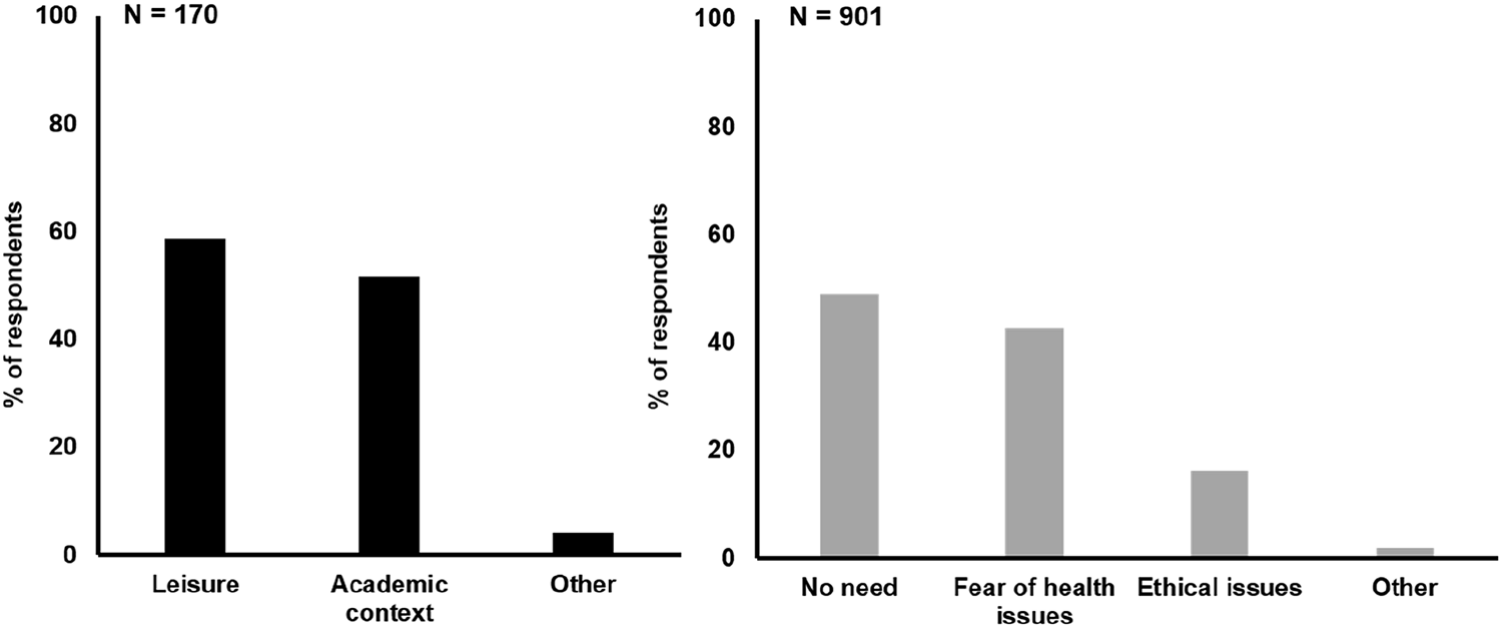
Motives for taking stimulants (*n* = 170) and not taking stimulants (*n* = 901) without prescription (relative frequencies; multiple answers possible)

Absolute and relative frequencies of respondents who endorsed statements on feigning ADHD and stimulant misuse are presented in Table 2. Twenty-four students (2.2%) reported to have considered feigning ADHD themselves, while 180 students (16.8%) knew someone who feigns ADHD. Furthermore, 611 students (57.1%) indicated that they think it is easy to feign ADHD, and 553 students (51.7%) assume that there are benefits to feign ADHD. Of the 553 respondents who indicated that feigning ADHD has benefits, 50.1% assumed benefits in the academic context (such as receiving any form of accommodation at university or support to increase work performance, e.g. allowance of different forms of exams, receiving extra time for assignments and exams, getting an own room for taking exams, exception from rules, entering support programs at university, etc.), 41.2% assumed access to stimulant medication, 39.4% assumed benefits in the social context (such as receiving attention from others, being pitied, having an excuse, more social support, privileges, or not taking responsibility for own behavior), 5.1% assumed financial support (e.g. special bursaries, subsidies, money for buying technical facilities for studying, money for entering treatment and support programs), and 8.1% of those participants assumed any other form of (not specified) benefits (multiple answers possible). Moreover, 724 students (68.0%) endorsed that prescription stimulants were also beneficial for someone not diagnosed with ADHD, and 170 students (15.9%) stated that they had taken stimulants themselves. Of those respondents who indicated to have ever taken stimulants without prescription, 58.3% reported to take them occasionally and 1.2% regularly, whereas 40.5% reported to have taken stimulants only once. Most of the people who took stimulants without prescription got access to the medication via peers (80.0%), or bought them on the black market (30.6%) (multiple answers possible). A smaller proportion received the stimulants from family members who were diagnosed with ADHD (11.8%) or from other sources (2.4%). Figure 1 presents the relative frequencies of motives that were reported for taking stimulants without prescription (*n* = 170) or for not taking stimulants without prescription (*n* = 901). The motives for taking stimulants concerned for more than half of the respondents leisure activities and the academic context. The most common motives for not taking stimulants regarded no perceived need (e.g. not necessary, no need, not interested, like to stay as they are, etc.) and concerns with health issues (e.g. addictive, physical and psychological consequences, damaging to own body, unpredictable effects on body, both short and long-term consequences feared, potential side effects, etc.). A lower number of students indicated ethical matters in this context (e.g. illegal behavior, not fair to others, having an undeserved advantage, responsibility towards society, intention to show good and honest behavior, etc.)

**Table 2:** Agreement to statements on feigning ADHD and stimulant misuse

Item		Agreement (of *n* = 1071)		
		% (relative)	*N*	%
*Feigning ADHD*				
Ever considered to feign ADHD?			24	2.2
Knowing someone who feigns ADHD?			180	16.8
Easy to feign ADHD?			611	57.1
Benefits to feign ADHD?			553	51.7
Benefits in academic context		50.1		
Access to stimulant medication		41.2		
Benefits in social context		39.4		
Financial support		5.1		
Other benefits		8.1		
*Stimulant misuse*				
Stimulants beneficial for someone not diagnosed with ADHD?			724	68.0
Ever taken stimulants without prescription?			170	15.9
*Frequency?*				
Once		40.5		
Occasionally		58.3		
Regularly		1.2		
*Source?*				
Peers		80.0		
Black market		30.6		
Family member with ADHD		11.8		
Other sources		2.4

## Discussion

The present study highlights that about half of the Dutch university students of the present sample assume benefits regarding the feigning of ADHD (52%) and express their confidence in the ease to feign ADHD (57%). The assumed benefits of feigning ADHD are of various types, going beyond getting access to stimulant medication. Such perceived advantages include most often accommodation at university to improve study performance (e.g. allowance of different forms of exams, and/or receiving extra time for assignments and exams), and benefits in the social interaction with family and friends, such as receiving attention from others, being pitied, or having an excuse for academic failure or occasional misbehavior. The fact that a substantial number of students perceive benefits from an ADHD diagnosis in the social context may indicate a low perceived stigma associated with ADHD, as the diagnosis of ADHD is seemingly not seen as a label that sets individuals apart and may result in depreciation. Instead, the label ADHD may even bring benefits in the way others have understanding for their situation and treat them. The risk of feigned ADHD among university students is not only shown by 2% of the sample who indicated to have ever considered feigning ADHD themselves, but also by the fact that almost 1 in 5 students (17%) knows someone who feigns ADHD.

Both, the positive attitudes towards the effects of prescription stimulants for people not diagnosed with ADHD as shown by 68% of the students and the substantial number of students (16%) who indicated to take stimulants without prescription confirm previous research that reported widespread stimulant misuse on university campuses in North America. Previous studies tried to characterize students who take stimulants without prescription. These studies found that in comparison to students not taking stimulants, stimulant misusers are more likely to have neuropsychological dysfunction (Wilens et al. 2017) and also more likely to endorse alcohol, drug, and substance use disorders. Furthermore, higher rates of psychiatric illness and general dysfunction has been reported for stimulant misusers (Wilens et al. 2016). In addition, positive associations were found between stimulant misuse and psychological distress and internal restlessness (Weyandt et al. 2009). Although findings are correlational and not causal in nature, it has been speculated that stimulant misuse is often motivated by the intention to improve cognitive performance in order to achieve higher grades at university (Blevins et al. 2017). In this respect, the self-reported motives for stimulant misuse of students in the present study are largely in line with the motives as reported in previous work, i.e. to improve cognitive and thus academic performance, but also for recreational use such as getting high (Rabiner 2013; Teter et al. 2006; Blevins et al. 2017; Barrett et al. 2005; Hartung et al. 2013).

Moreover, research has shown that students reporting prescription stimulant misuse have a lower risk perception of stimulant drugs as compared to students not reporting misuse (Blevins et al. 2017). It was found that students who regularly take stimulants without prescription are less aware of the addictive character of stimulants as well as the serious consequences that can emerge from misuse, including psychosis, seizures, cardiovascular events, and even sudden death (Lakhan and Kirchgessner 2012). In this context, it appears relevant that also in the present study one of the primary reasons for students not taking stimulants were concerns regarding health issues. Finally, also the media may play a role in the risk–benefit perception of stimulant misuse, as the media seem to tend to report more often the benefits of prescription drugs for neuroenhancement (in 95% of all media reports on this topic), but often fails to mention its possible risks and side effects (in only 58% of the respective media reports) (Partridge et al. 2011).

In conclusion, this is the first study providing data about the attitudes and opinions of university students in the Netherlands towards feigning ADHD and misusing stimulant drugs. The present results reveal that Dutch university students have a liberal view towards the feigning of ADHD and alarmingly high rates of stimulant misuse among Dutch students. The diagnostic evaluation of ADHD among university students should therefore be performed with caution, and a careful exploration of the credibility of their symptoms is warranted. While earlier consensus reports and position papers already advocated the necessity to include validity measures of self-reports and performance in all neuropsychological evaluations (Bush et al. 2005; Heilbronner et al. 2009), irrespectively of the context, recent research gave more explicit advice regarding the assessment of adult ADHD. For example, the recent 20-year update of the Slick criteria for the assessment of malingered neuropsychological dysfunction (Sherman et al. 2020) stressed specifically the substantial external incentives that may motivate people to feign ADHD, as well as the high rates (up to 50%) of noncredible responses and performance at clinical evaluation of ADHD in the university setting. Thus, the use of measures for symptom and performance validity in routine clinical assessment of adult ADHD seems to be advisable.

Furthermore, it could be speculated that campaigns aiming to inform students about the addictive character and serious consequences that can emerge from stimulant misuse may be helpful. The benefits of such campaigns should be addressed in future research.

## Limitations and future directions

This study has to be seen in the context of several limitations. Of note, the data are based on self-report only, which may be prone to bias. For example, careless or inattentive responding have been observed in a non-trivial number of participants taking part in questionnaire studies (e.g. see Oppenheimer et al. 2009 for the occurrence and suitable detection strategies). Furthermore, in this particular study, the sensitive topic of feigning ADHD and misusing stimulants may have triggered biased responses towards positive impression management and hesitations of admitting illegal behavior. The data must therefore be interpreted with caution as to how much they represent real prevalence rates among this student population. Next to including detection measures for invalid response styles (including carelessness, ‘faking good’, or ‘faking bad’), It would be worthwhile to combine self-reports as derived from surveys with more objective data as derived from individual assessments, e.g. university students at clinical evaluation for ADHD failing symptom and/or performance validity assessment (see for example Harrison and Armstrong 2020).

Furthermore, it must be stressed that the present study may contribute to uncovering opinions and attitudes towards feigning ADHD, but this should not be confused with the frequency of feigning ADHD among students. For example, even though it was presented how many respondents considered to feign ADHD, no data are available on the number of students actually attempting to feign ADHD. Also, the 17% of the students that report to know someone who attempted to feign ADHD does not inform on the frequency since it remains unknown to what extend students are acquainted with each other (and referring to the same persons).

Finally, future research needs to determine differences in the opinions and attitudes on this issue across faculties and study years, and is also advised to take special study periods into consideration. For example, it could be speculated that time periods exposing students to acute stress (e.g. exam periods) or revealing academic failure (e.g. post exam periods) may change opinions towards use of stimulants and potentially feigning ADHD.

## Data Availability

The data that support the findings of this study are available from the corresponding author upon reasonable request.
